# Precision medicine for adjuvant chemotherapy of resected colorectal cancer

**DOI:** 10.1002/ags3.12397

**Published:** 2020-12-08

**Authors:** Yuji Miyamoto, Yukiharu Hiyoshi, Hiroshi Sawayama, Ryuma Tokunaga, Hideo Baba

**Affiliations:** ^1^ Department of Gastroenterological Surgery Graduate School of Medical Sciences Kumamoto University Kumamoto Japan

**Keywords:** adjuvant chemotherapy, colorectal cancer, precision medicine, predictive marker, prognostic marker

## Abstract

Colorectal cancer (CRC) is the most common cancer and the second leading cause of cancer death in Japan. Surgical resection is the only curative option for localized disease. However, undetectable micrometastases remaining after curative surgery may cause disease recurrence. Adjuvant chemotherapy aims to eradicate these micrometastases to improve the cure rate. Unfortunately, few reliable prognostic and predictive markers are available that identify patients at high risk for CRC during early‐stage disease. However, promising biomarkers may become available in the near future. Such biomarkers provide information for stratifying a patient's risk and for selecting the optimal treatment. Here, we provide an overview of current relevant prognostic and predictive biomarkers applicable to adjuvant treatment of early‐stage CRC and focus on the future of this field.

## Graphical abstract

During the last decade, numerous efforts have been made to develop precision medicine for patients with early colorectal cancer. We summarize prognostic and predictive biomarkers for adjuvant treatment of early colorectal cancer.

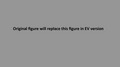

## 1 INTRODUCTION

Colorectal cancer (CRC) is the most common cancer and the second leading cause of cancer death in Japan, with more than 130 000 new cases diagnosed each year.^1^ Surgical resection is the only curative option for localized disease, and its outcome is most closely associated with the extent of disease upon presentation. However, disease recurrence is caused by undetectable micrometastases that persist after curative surgery. Adjuvant chemotherapy aims to eradicate these micrometastases to improve the cure rate.

Disease stage is the most important pathological prognostic factor after surgery. For example, the Japanese Multicenter Registry reported recurrence rates after potentially curative surgery of approximately 15% and 32% for stages II and III CRC, respectively.^2^ Furthermore, a phase III study of patients treated in Japan found that the 5‐year disease‐free survival (DFS) rates of patients with stages IIIA, IIIB, and IIIC were 90.4%, 74.1%, and 58.9%, respectively.^3^

As of early 1990, 5‐fluorouracil (5‐FU)‐based regimens were generally accepted as a standard adjuvant regimen for patients with stage III and selected patients with stage II colon cancer with 5%‐10% improvement in absolute survival.^4^, ^5^, ^6^, ^7^ Furthermore, the addition of oxaliplatin lengthens DFS and OS of patients with stage III CRC.^8^, ^9^, ^10^ The benefit of these adjuvant chemotherapies is most clearly demonstrated by the approximately 30% relative risk of recurrence of stage III disease.^11^, ^12^ However, the importance of adjuvant chemotherapy in the treatment of stage II disease is unclear. Several clinical trials found that adjuvant chemotherapy of resected stage II colon cancer confers a minimal benefit upon OS.^3^, ^13^, ^14^

The survival data summarized above raise obvious and important questions as follows: (a) How can we effectively identify patients with stage II CRC at high risk of recurrence who will potentially benefit from adjuvant chemotherapy? (b) Most patients with stage III CRC recieve adjuvant chemotherapy despite a >50% surgical cure rate. Are they overtreated? (c) Approximately 30% of patients with stage III CRC who undergo adjuvant therapy experience recurrence. Are these patients candidates for additional therapy?^2^, ^15^

In this era of precision medicine, molecular characterization of tumors is essential for selecting a therapeutic strategy. Consequently, the identification and standardization of prognostic and predictive molecular biomarkers for cancer are becoming increasingly relevant. Here, we summarize knowledge of candidate prognostic and predictive biomarkers for adjuvant treatment of stages II and III CRC (Table 1), with a focus on the future of this critically important field.

**TABLE 1:** Summary of studies

Study	Year	N	Stage	Marker	Prognostic value		Predictive value		Reference
					DFS, RFS, RFI	OS	5FU‐base	Dublet	
					HR (RR) (95% CI)	HR (95% CI)	Predictive value	Predictive value	
Tumor budding									
SACURA	2019	991	II	BD3 vs BD1	2.57 (1.69‐3.91)	N/A	Possible	N/A	18
MSI									
Ribic et al	2003	570	II‐III	MSI‐H vs MSS or MSI‐L	N/A	0.61 (0.38‐0.96)	Yes	N/A	23
QUASAR	2011	1913	II‐III	dMMR vs pMMR	0.53 (0.40‐0.70)	N/A	No	N/A	26
Sinicrope et al	2011	2141	II‐III	dMMR vs pMMR	0.80 (0.64‐0.99)	0.79 (0.64‐0.99)	No	N/A	27
KRAS									
RASCAL II	1998		I‐IV	Codon 12 (p.Gly12Val)	1.30 (1.09‐1.54)	1.29 (1.08‐1.55)	N/A	N/A	36
PETACC3	2010	1299	II‐III	KRAS status	NS	NS	N/A	N/A	38
CALGB89803	2009	508	III	KRAS status	NS	NS	N/A	N/A	39
QUASAR	2011	1583	II‐III	KRAS status	1.40 (1.12‐1.74)	N/A	No	N/A	26
PETACC8, N0147	2017	4189	III	KRAS mutation in MSS	1.60 (1.60‐1.83)	1.52 (1.29‐1.79)	N/A	N/A	30
BRAF									
PETACC3	2010	1307	II‐III	BRAF status	NS	1.66 (1.15‐2.40)	N/A	N/A	38
CALGB89803	2012	506	III	BRAF status	NS	1.66 (1.05‐2.63)	N/A	Possible	43
QUASAR	2011	1584	II‐III	BRAF status	NS	N/A	No	N/A	26
NSABP C‐07	2012	2299	II‐III	BRAF status	NS	1.46 (1.20‐1.79)	N/A	N/A	42
TP53									
Elsaleh et al	2001	891	III	TP53 mutation	N/A	N/A	Yes	N/A	48
TP53‐CRC	2005	3583	I‐IV	TP53 mutation in distal CC	2.52 (1.28‐4.93)	N/A	N/A	N/A	49
NSABP C01‐04	2003	706	II‐III	TP53 mutation	1.49 (1.12‐1.98)	NS	No	N/A	50
Elsaleh et al	2000	388	III	TP53 mutation	N/A	NS	No	N/A	51
Consensus molecular subtype									
NSABP C07	2016	382	III	CMS2‐enterocyte	N/A	N/A	N/A	Possible	54
Immunoscore									
Pages et al		1562	I‐III	Immunoscore (high vs low)	0.502 (0.394‐0.639)	0.560 (0.431‐0.728)	N/A	N/A	59
Circulating tumor DNA									
Tie et al	2016	230	II	ctDNA positive vs negative	14 (6.8‐28)	N/A	Potential value as a real‐time marker		62
Tie et al	2019	100	III	ctDNA positive vs negative	7.5 (3.5‐16.1)	N/A	Potential value as a real‐time marker		63
Reinert et al	2019	130	I‐III	ctDNA positive vs negative	7.2 (2.7‐19.0)	N/A	Potential value as a real‐time marker		64
Gene expression signature									
QUASAR	2010	1436	II	RS (continuous)	1.43 (1.11‐1.83)	N/A	No	N/A	68
GALGB9581	2013	690	II	RS (continuous)	1.68 (1.18‐2.38)	N/A	N/A	N/A	69
SUNRISE	2016	630	II‐III	RS (continuous)	2.05 (1.47‐2.86	N/A	N/A	N/A	70
NSASBP C‐07	2013	892	II‐III	RS (continuous)	1.96 (1.50‐2.55)	N/A	N/A	no	71
Sazarl et al	2011	206	I‐III	ColoPrint (high vs low)	2.69 (1.41‐5.14)	N/A	N/A	N/A	72
Kopetz et al	2015	416	II	ColoPrint (high vs low)	2.16 (1.28‐3.65)	N/A	N/A	N/A	73
Maak et al	2013	135	II	ColoPrint (high vs low)	4.28 (1.36‐13.50)	N/A	N/A	N/A	74
CDX2 expression									
Dalerba et al	2016	314	II‐III	CDX2 expression negative	2.42 (1.36‐4.29)	N/A	Predictive value in stage II		78

Note

BD, budding; CC, colon cancer; CI, confidence interval; ctDNA; circulating tumor DNA; DFS, disease‐free survival; dMMR, deficient mismatch repair; HR, hazard ratio; MSI‐H, high microsatellite instability; MSI‐L, low microsatellite instability; MSS, microsatellite‐stable; NS, no significant; N/A, not applicable; OS, overall survival; pMMR, proficient mismatch repair; RFI, relapse‐free interval; RFS, relapse‐free survival; RR, risk ratio; RS, recurrence score.

## 2 CLINICOPATHOLOGICAL VARIABLES

Clinicopathological variables are essential for selecting chemotherapy for patients with stage II colon cancer. Certain clinicopathological factors identify patients at high‐risk stage II for colon cancer, such as T4 primary, high‐grade/poorly differentiated histology, lymphatic/vascular invasion, perineural invasion, bowel obstruction or perforation, indeterminate/positive margins, or <12 lymph nodes examined.^16^ Patients with these tumors are considered candidates for adjuvant chemotherapy with 5‐FU‐based chemotherapy, FOLFOX, or CAPEOX. However, an analysis of a database comprising 24 847 patients who underwent resection for colon cancer did not experience a survival benefit conferred by 5‐FU‐based adjuvant chemotherapy, even for those with high‐risk stage II colon cancer.^17^ In this study, there was no significant survival benefit for patients with stage II colon cancer with or without poor prognostic features.

The benefit of oxaliplatin as adjuvant therapy for patients with high‐risk stage II colon cancer was evaluated by the MOSAIC trial.^8^ This trial found a trend toward improved 5‐year DFS with FOLFOX4 (82% vs 75%, HR = 0.72, 95% CI = 0.50‐1.02) of patients with stage II with high‐risk tumors (T4, tumor perforation, bowel obstruction, poorly differentiated tumor, venous invasion, or <10 lymph nodes examined in the surgical specimen).^8^ The OS rates of both groups were not significantly different (85% vs 83%, HR = 0.91, 95% CI = 0.61‐1.36, *P* = .65). These results do not support the conclusion that oxaliplatin‐based therapy benefits patients with high‐risk stage II disease, because the MOSAIC trial lacked a control group that underwent surgery alone.

### 2.1 Tumor budding

Another finding may contribute new insights to postoperative adjuvant therapy for stage II CRC. The clinical value of the tumor budding status as a tumor‐associated prognostic factor was addressed by the prospective, randomized controlled SACURA trial^3^ This trial evaluated the superiority of adjuvant treatment with oral tegafur‐uracil compared with surgery alone for stage II colon cancer.^18^

Tumor budding is defined as a single tumor cell or a cell cluster consisting of ≤ 4 tumor cells.^19^ The International Tumor Budding Consensus Conference was held to reach an agreement on an international, evidence‐based standardized scoring system for tumor budding in CRC.^19^ High tumor budding status is significantly associated with a lower 5‐year recurrence‐free survival (RFS) rate, and multivariate analyses of RFS revealed that budding status is an independent prognostic factor.^18^ Interestingly, there was a tendency for the beneficial effect of Tegafur‐uracil in patients with highly budding tumors (moderate budding grade [BD]: HR = 0.84, 95% CI = 0.53‐1.33; high BD: HR = 0.72, 95% CI = 0.41‐1.27), but not in patients with low‐budding tumors (low BD: HR = 1.14, 95% CI = 0.60‐2.16).

These results suggest that tumor budding may serve as a useful marker to enhance decision‐making for optimizing adjuvant chemotherapy, which is supported according to its predictive significance when applied to patients with stage II colon cancer. A randomized study (JCOG 1805) is evaluating the predictive value of budding for adding adjuvant chemotherapy to treat patients with stage II CRC in Japan. JCOG 1805 comprises 1680 patients who will be randomly allocated to groups receiving capecitabine, CAPEOX, or surgery alone, with a primary endpoint of RFS and a secondary endpoint of DFS and OS.

## 3 GENETIC ALTERATIONS

Recent studies summarized below focus on identifying specific genomic mutations that serve as predictive biomarkers of adjuvant chemotherapy after surgical treatment that can be employed to individualize treatment. Several key mutations in CRC are important for its initiation, progression, metastasis, and response to therapeutics.

### 3.1 Microsatellite instability (MSI)

Microsatellite instability is caused by a deficiency in DNA mismatch repair (MMR), resulting in the accumulation of mutations in DNA. DNA‐MMR deficiency is detected in approximately 15% of sporadic CRCs.^20^ A germline mutation that inactivates MMR genes may lead to Lynch syndrome, which is a common hereditary disorder that predisposes patients to CRC. Young age and a positive family history of CRC are risk factors for Lynch syndrome. MSI/dMMR tumors are characteristically located on the right side of the colon, exhibit mucinous histology with tumor‐infiltrating lymphocytes, and are most frequently diagnosed as stage II.^21^ Furthermore, most studies show that MSI/dMMR is an independent favorable prognostic factor of survival of patients with CRC.^22^, ^23^, ^24^

Ribic et al^23^ published the first report of differential benefit conferred by fluorouracil‐based adjuvant chemotherapy upon patients with stages II and III colon cancers with microsatellite‐stable and MSI‐low tumors compared to those with MSI‐high tumors. These findings are consistent with those of a systematic review of seven studies that stratified survival of patients with CRC according to MSI status.^25^ In contrast, two analyses confirm the prognostic significance of dMMR but not its predictive capability.^26^, ^27^ The predictive ability of MSI for 5‐FU‐based chemotherapy is uncertain because of the controversial findings among studies aimed to predict the role of MSI status in the response to chemotherapy. The ESMO guidelines state that MSI/MMR status is not useful as guidance for making treatment decisions,^28^ reflecting the heterogeneity of data for potential predictive values.

Further analysis of patients included in the MOSAIC trial points to a potential benefit of adding oxaliplatin to adjuvant 5‐FU/leucovorin administered to patients with MSI‐high stage III colon cancer.^29^ Although this study is underpowered, oxaliplatin is associated with a discernible, but not statistically significant, decrease in mortality of patients with dMMR tumors (HR = 0.41, 95% CI = 0.16‐1.07, *P* = .069). Further, the pattern suggests that more benefit is conferred by oxaliplatin upon the dMMR than the pMMR population (HR = 0.91, 95% CI = 0.72‐1.15, *P* = .43).

### 3.2 KRAS

KRAS is a proto‐oncogene encoding a 21‐kDa GTP‐binding protein that regulates cellular responses to numerous extracellular stimuli. KRAS mutations represent 15%‐37% of early‐stage tumors^30^, ^31^ and 45% of metastatic colorectal tumors.^32^ Furthermore, mutant KRAS constitutively activates downstream components of the PI3K/Akt pathway and MAPK pathways.^33^ In the metastatic setting, KRAS mutational status is recognized as a predictive biomarker of resistance to anti‐EGFR antibody therapy.^34^, ^35^ However, the roles of the KRAS mutational status as a prognostic and predictive biomarker in the adjuvant setting are controversial.

The RASCAL I/II studies found that KRAS mutational status (particularly the codon 12 glycine‐to‐valine mutation) is associated with poor prognosis of patients with stage II or III disease.^33^, ^36^, ^37^ In contrast, phase III translational studies (PETACC3 and CALGB 89803) found that the KRAS mutational status does exert a significant prognostic impact on CRC treated with adjuvant 5‐FU‐based chemotherapy.^38^, ^39^ Moreover, the QUASAR trial determined the KRAS mutational status of patients with 1913 CRC who were randomly assigned 5‐FU/LV chemotherapy or no chemotherapy. These data further showed that a KRAS mutation predicts failure to respond to 5‐FU/LV chemotherapy.^26^

A pooled analysis of the PETACC‐8 and N0147 trials of an oxaliplatin regimen found that a KRAS mutational status is a prognostic factor of stage III disease.^30^ In this cohort of 4411 patients with stage III colon cancer, KRAS exon‐2 mutations were identified as independent predictors of shorter time to recurrence (HR = 1.60, 95% CI = 1.60‐1.83, *P* < .01) and OS (HR = 1.52, 95% CI = 1.29‐1.79, *P* < .01) among patients with stage III microsatellite‐stable (MSS) tumors. These results suggest that KRAS mutational status may serve as a prognostic marker in the adjuvant oxaliplatin‐based chemotherapy. In contrast, despite the value of *KRAS* mutational status as prognostic markers in the adjuvant setting, most studies did not find a significant association between KRAS mutational status and the response to standard chemotherapy.

### 3.3 BRAF

BRAF is the principal downstream effector molecule of RAS signaling in the RAS‐RAF‐MEK/ERK kinase signal transduction pathway.^40^ A large population‐based study found that the BRAF V600E mutation occurs in 5% of MSS tumors and in 50% percent of MSI‐H tumors.^41^ In the adjuvant setting, BRAF mutational status is a valid prognostic marker.^38^, ^41^, ^42^ The randomized adjuvant chemotherapy trials CALGB 89 803 and PETACC‐3 assessed the prognostic role of BRAF mutational status patients with stages II or III CRC.^38^, ^43^ The data show that patients with BRAF‐mutated tumors experienced significantly shorter OS compared with those with BRAF wild‐type tumors.

The QUASAR trial, which evaluated BRAF mutational status as predictive markers for 5‐FU/LV chemotherapy, was unable to detect an association between BRAF mutational status and tumor response to 5‐FU/LV chemotherapy.^26^ Further, the results of the NSABP C‐07 trial did not detect a significant association between BRAF mutational status and the benefit of oxaliplatin.^42^ Despite their established role as prognostic markers, BRAF mutational status may not serve as predictive markers of adjuvant chemotherapy.

### 3.4 TP53

TP53 encodes a tumor suppressor that initiates cell cycle arrest, apoptosis, DNA repair, and the inhibition of angiogenesis.^44^ TP53 activates the transcription of numerous downstream target genes by binding to their regulatory sequences. Genetic alterations of TP53, which are frequent events in colon cancer, have been studied extensively to evaluate their associations with patients' prognoses and responses to adjuvant chemotherapy. However, certain results conflict, likely because of the complex biology of TP53 function and the different methodologies used to detect TP53 alterations.^45^, ^46^, ^47^

A systematic review found that abnormal TP53 is associated with an increased relative risk of death, regardless of whether immunohistochemistry or DNA mutation analysis was used.^46^ In clinical studies of adjuvant chemotherapy‐treated and untreated groups, patients with stage III CRC whose tumors overexpressed TP53 experienced significantly shorter survival following 5‐FU‐based chemotherapy than patients whose tumors did not express TP53 with detectable alterations.^48^, ^49^ However, other studies of patients with colon cancer failed to demonstrate correlations between TP53 alterations and the benefit of adjuvant therapy.^50^, ^51^

## 4 CONSENSUS MOLECULAR SUBTYPES (CMS) CLASSIFICATION

The identification of a molecular classification that can predict therapeutic responses to adjuvant chemotherapy is a major goal of cancer research. Numerous studies use gene expression profiling to identify molecular CRC subtypes. However, these classifications reveal only superficial similarities. To resolve these inconsistencies, the international CRC Subtyping Consortium compared six independently transcriptomic‐based subtyping systems and identified a consensus gene expression‐based subtyping classification system for CRC. This resulted in the definitions of CMS that categorize most tumors into one of four subtypes.^52^ The CMS classification provides insights into the biological understanding of each subtype.^53^

CMS1 is enriched in MSI tumors that activate immune cells. CMS2 reflects the classical enterocyte subtype encompassing typical WNT/MYC‐driven tumors with epithelial characteristics, whereas CMS3 is enriched in KRAS‐mutated tumors with activation of metabolic pathways. CMS4 has mesenchymal features similar to those of cancer stem‐like cells and includes high stromal content and activation of TGF‐β and VEGFR pathways.

Recent work shows that colorectal subtypes can be used to predict responses to therapy. The NSASBP C‐07 study re‐estimated the expression levels of 72 genes to determine molecular subtypes of patients with stage II or III colon cancer and their associations with prognosis and interactions with oxaliplatin therapy.^54^ The CMS4 subtype (or the stem‐like subtype) is associated with poor prognosis of patients treated with both 5FU‐based and oxaliplatin‐based chemotherapy, regardless of clinical stage. This analysis found that only patients with stage III CMS2 tumors (or the enterocyte subtype) benefitted from oxaliplatin‐based adjuvant chemotherapy compared with fluorouracil alone. Although this was not found in the validation cohort of this study using CRC Assigner (CRCA) subtypes, this result warrants additional investigations of external series. These associations between the CMS classification and chemosensitivity (5FU or oxaliplatin) were confirmed by a study conducted in vitro.^55^

## 5 IMMUNOSCORE

Accumulating evidence suggests that the tumor microenvironment is required for disease progression and tumor resistance to chemotherapy,^56^, ^57^ such that the assessment of tumor‐infiltrating lymphocytes for prognostication and prediction of benefit from adjuvant chemotherapy is critically important.^58^ The Immunoscore is a scoring system based on the densities of CD3^+^ and CD8^+^ T cells at the tumor center and the invasive margin, which is determined using immunohistochemistry and quantified using digital pathology.

The Immunoscore was defined in a large international validation study of 2681 patients with stages I‐III colon cancer.^59^ Patients with a high Immunoscore are at lowest risk of recurrence (HR, high vs low Immunoscore = 0.20, 95% CI = 0.10‐0.38, *P* < .0001). These findings were independently confirmed using internal and external validation sets. Among patients with stage II colon cancer, the Immunoscore accurately assessed relapse risk regardless of MSI status and identified patients for whom only surgery would be a sufficient treatment option. Furthermore, the ability of the Immunoscore to predict OS was superior to that of existing tumor‐risk parameters. Interestingly, the immune infiltrate varied widely between patients; 21% of patients with MSS tumors had high Immunoscores. These data suggest that factors such as characteristics of tumors, the microenvironment, and genetic and epigenetic alterations may affect the quality and density of the immune infiltrate.

Recently, the IDEA France cohort study evaluated the associations between the Immunoscore and DFS after 3 or 6 months of oxaliplatin‐based adjuvant chemotherapy administered to patients with stage III colon cancer.^60^ Furthermore, patients with an intermediate or high Immunoscore in the clinical low‐ (T1‐T3, N1) and high‐risk (T4 and/or N2) groups derived a significant benefit from the 6‐month mFOLFOX6 regimen compared with the 3‐month treatment.

## 6 CIRCULATING TUMOR DNA

The analysis of circulating tumor DNA (ctDNA) detects minimal residual disease and is associated with recurrence of CRCs. This technology predicts which patients require adjuvant therapy because of residual tumor subsequent to curative resection. For example, ctDNA analysis of blood collected after surgery for primary or metastatic CRC effectively identifies patients with residual disease who are predicted to experience disease recurrence.^61^ The study estimated the average half‐life of ctDNA after complete resection as 114 min. The short half‐life of ctDNA makes it an ideal dynamic biomarker of tumor burden that can be monitored after surgery.

Other studies report consistent results for patients with stage II or III CRC with ctDNA, after curative surgery, who experience poor outcomes despite adjuvant chemotherapy.^62^, ^63^, ^64^ These results suggest that ctDNA analysis may help physicians to make decisions to add or omit chemotherapy after curative resection for patients with stage II or III CRC.

Analysis of ctDNA has potential impact as a real‐time marker of the effectiveness of adjuvant therapy and may contribute to the rapid development of novel strategies for administering adjuvant therapies.^65^ Several randomized controlled studies are currently ongoing (NCT04068103, NCT03748680).

## 7 GENE EXPRESSION

### 7.1 Gene expression signature

Gene expression is intimately linked to cellular phenotype and tumor behavior and is extensively used to identify biologically homogeneous subtypes of a disease. However, with a few exceptions, many single‐gene expression markers make it difficult to predict the risk of recurrence in patients with CRC. Therefore, studies were conducted to improve predictive accuracies using combinations of multiple biomarkers. Prognosis predictions using gene expression signatures (OncotypeDX, Coloprint, GeneFX, OncoDefender‐CRC, and ColonPRS) are currently available for stages II and III CRC.

OncotypeDX is a 12‐gene RT‐PCR assay to identify genes that predict recurrence and treatment effects of stages II‐III colon cancer. After its clinical significance was initially introduced in an analysis of breast cancer,^66^ the assessment was applied to patients with CRC. For example, one study assessed the association between colon cancer recurrence and expression of multiple genes detected using paraffin‐embedded tumor tissues acquired from patients with stage II or III colon cancer treated in four independent trials of adjuvant therapy that included surgery‐alone and 5‐FU‐adjuvant chemotherapy arms.^67^ Among an initial 761 candidate genes, 12 were selected according to their independent association with recurrence in each of the studies. The analysis, which includes five reference genes, found that seven and six genes are significantly associated with recurrence and treatment benefit, respectively.

The prognostic accuracy of Oncotype DX was validated in an analysis of data from the prospective QUASAR trial^68^ of stage II colon cancer. Among the 711 patients enrolled in the surgery‐alone arm of the study, the seven‐gene recurrence score was significantly associated with recurrence risk 3 years after surgery (*P* = .004). Recurrence risks at 3 years were 12%, 18%, and 22% for predefined low‐, intermediate‐, and high‐recurrence risk groups, respectively. Additional validation involved a separate analysis of data of the CALGB 9581 trial^69^ of stage II colon cancer and the SUNRISE study^70^ of stages II‐III colon cancer.

The predictive value of this treatment score could not be validated using the QUASAR sample. An additional prospective study was designed for clinical validation of patients in the NSASBP C‐07 trial with stages II‐III colon cancer who received 5FU alone or oxaliplatin‐based therapy.^71^ This study found that Oncotype DX is not a predictor of oxaliplatin treatment efficacy and therefore cannot identify patients for whom oxaliplatin is not beneficial. However, this study provides evidence that patients with higher recurrence scores may derive an absolute benefit from oxaliplatin vs those with a low recurrence score.

ColoPrint is an 18‐gene expression signature designed to predict disease relapse in patients with early‐stage CRC. Salazar et al first reported the development and validation of this expression signature based on the data for a training set of 188 patients with CRC who underwent surgery.^72^ In another set of 206 patients with stages I‐III CRC, the 5‐year DFS rates of the low‐risk and high‐risk groups were 87.6% and 67.2%, respectively (HR = 2.5, 95% CI = 1.33‐4.73, *P* = .005). Coloprint identifies 60% of patients with stage III colon cancer at low risk of recurrence, which may be safely managed without adjuvant chemotherapy.^72^ Furthermore, several validation studies of patients with stage II colon cancer were conducted.^73^, ^74^ For example, a study of 416 patients with stage II colon cancer^73^ found that ColoPrint identifies 37% of patients at high risk, with a 21% 5‐year risk of relapse, whereas low‐risk patients (67%) have a 5‐year risk of relapse of 10% (HR = 2.16, 95% CI = 1.28‐3.65, *P* = .004). The analysis of patients who did not receive adjuvant treatment achieved the same prognostic power as the analysis of all patients (HR = 2.38, *P* = .008). A prospective observational study is ongoing to examine risk stratification using ColoPrint analysis of 1200 patients with stages II‐III colon cancer, including 575 patients with stage II disease (NCT00903565).

### 7.2 CDX2 expression

CDX2 is a highly sensitive and specific marker of adenocarcinomas of intestinal origin.^75^ CDX2 is a tumor suppressor, and its expression is frequently downregulated in CRC.^76^ Furthermore, colon cancers that lack detectable CDX2 expression are associated with advanced disease stage, poor differentiation, vascular invasion, BRAF mutations, and the CpG island methylator phenotype.^77^

A more recent study identified CDX2 as a candidate biomarker through a bioinformatics approach that included 2466 human gene expression array experiments involving 28 independent GEO data‐series.^78^ The results show that CDX2 expression is a prognostic and predictive biomarker of early‐stage colon cancer. This study found that loss of detectable CDX2 expression is associated with significantly shorter DFS, OS, and disease‐specific survival compared with CDX2‐positive CRCs. These findings are consistent with the evaluation of CDX2 protein expression of a validation data set. Among patients with stage II disease, treatment with adjuvant chemotherapy is significantly associated with longer DFS of the CDX2‐negative subgroup, although it is not significantly associated with longer DFS of the CDX2‐positive subgroup. Among patients with stage III disease, treatment with adjuvant chemotherapy is significantly associated with longer DFS of the CDX2‐negative and CDX2‐positive subgroups. A test for an interaction between the biomarker and treatment outcomes indicates that in stages II and III disease, there is a significant benefit associated with adjuvant chemotherapy among CDX2‐negative patients than among CDX2‐positive patients.

These results suggest that CDX2 expression serves a prognostic biomarker for stages II and III colon cancer that may be effectively treated using adjuvant chemotherapy. Although encouraging, these results are derived from retrospective analyses of patient cohorts and pooled data sets, and therefore require validation by prospective randomized trials before CDX2 expression can be incorporated as a biomarker into routine clinical practice.

## 8 FUTURE PERSPECTIVES

The remarkable progress in developing artificial intelligence (AI) technologies such as machine learning and deep learning enables multimodal analyses of big omics data. AI can therefore be applied to developing a platform to conduct multimodal analyses of biomarker complexity to potentially accelerate the realization of the promise of precision medicine. For example, machine learning approaches using radiomic features predict the response to chemotherapy in neoadjuvant settings.^79^, ^80^, ^81^ Furthermore, other investigators analyzed machine‐learning techniques combining with molecular‐based parameters, which established clinicopathological features to identify the prognostic values of biomarkers.^82^, ^83^

The next promising field is the microbiota. The gut microbiota affects immunity, metabolism, and tissue development.^84^, ^85^, ^86^ Recently acquired evidence suggests that specific microbiomes such as those comprising *Fusobacterium* are associated with the response to chemotherapy of patients with gastrointestinal cancers.^87^, ^88^ In vitro studies show that the combination of *Fusobacterium* and tumor cells influences the trend of chemoresistance to 5‐FU and oxaliplatin.^87^, ^89^, ^90^ Moreover, *Fusobacterium nucleatum* induces chemoresistance of CRC cell lines via the autophagy pathway.^87^ Furthermore, a large population of *F. nucleatum* is associated with high risk of CRC recurrence, and the size of the population of *F. nucleatum* may serve as a prognostic marker of patients' outcomes.

Once the intestinal microbiota that improves the therapeutic effect of chemotherapy can be identified, this knowledge can be applied to postoperative adjuvant chemotherapy. Accordingly, patients with CRC with a high number of *F. nucleatum* may be treated with a combination of adjuvant chemotherapy and antimicrobials that target *F* *. nucleatum* with or without an inhibitor of autophagy.

Immune checkpoint inhibitors are widely accepted as indicated by their acceptable safety data and efficacy for treating refractory MSI‐positive metastatic CRC.^34^, ^91^ This level of efficacy is applicable to the adjuvant setting. The combination of immune checkpoint inhibitors with standard adjuvant chemotherapy may improve the prognosis of patients with stage III colon cancer with MSI or dMMR tumors. The current ATOMIC phase III randomized controlled trial, which compares the use of atezolizumab, a PD‐L1 inhibitor, in combination with FOLFOX vs FOLFOX alone, for the treatment of stage III CRC with dMMR tumors, may provide further evidence of the efficacy of such a therapeutic strategy (NCT02912559).

The next challenge is to expand immunotherapy to a broader class of tumors, including MSS cancers. For example, POLE mutations occur in 1% of patients with colon cancers, although the incidence ranges between 8% and 10% in those aged <50 years.^92^ Accordingly, the current phase III POLEM trial is designed to investigate the use of avelumab plus fluoropyrimidine‐based chemotherapy as adjuvant treatment for stage III colon cancers with dMMR or POLE mutations.^93^ Hopefully, these approaches will further personalize the treatment options in the adjuvant setting of CRC.

The regular use of aspirin after a diagnosis of colon cancer is associated with a superior clinical outcome. Interestingly, this effect of postdiagnosis aspirin use on survival appears to differ according to the *PIK3CA* mutational status.^94^, ^95^ A recent study shows that the association of aspirin use with CRC survival is significant for patients with CD274‐low tumors vs CD274‐high tumors.^96^ The ongoing Add‐Aspirin phase III, multicenter, double‐blind, placebo‐controlled randomized trial aims to determine the effects of aspirin on patients with CRC who have undergone potentially curative treatment.^97^ Its available feasibility results indicate that aspirin is well tolerated after radical cancer therapy.^98^ Another phase III trial is ongoing in Japan (JCOG1503C).^99^

## 9 CONCLUSIONS

During the last decade, numerous efforts have been made to develop precision medicine for patients with early colon cancer; however, biomarker‐guided adjuvant treatment options are limited. Furthermore, novel drugs with specific targeting activity are not effective for treating patients with early colon cancer. The only standardized and efficacious treatment is 5‐FU plus oxaliplatin‐based chemotherapy. Effective molecular biomarkers are therefore required to identify optimal treatment strategies for managing patients who will benefit from adjuvant chemotherapy. Among these biomarkers, MMR status, Immunoscore, CDX2, and ctDNA, as well as others, will help predict a specific prognosis and response to adjuvant chemotherapy of patients with CRC. Although there are numerous concerns over the accuracies of the studies of most cancer treatments, we are rapidly advancing along the path leading to improving a cancer patient's prognosis.

## DISCLOSURE

The authors declare no conflict of interest.
